# Anti‐CD20 monoclonal antibody therapy in postpartum women with neurological conditions

**DOI:** 10.1002/acn3.51893

**Published:** 2023-09-07

**Authors:** Annika Anderson, William Rowles, Shane Poole, Ayushi Balan, Carolyn Bevan, Rachel Brandstadter, Andrea I. Ciplea, Joanna Cooper, Michelle Fabian, Thomas W. Hale, Dina Jacobs, Mihir Kakara, Kristen M. Krysko, Erin E. Longbrake, Jacqueline Marcus, Pavle Repovic, Claire S. Riley, Andrew R. Romeo, Alice Rutatangwa, Timothy West, Kerstin Hellwig, Sara C. LaHue, Riley Bove

**Affiliations:** ^1^ UCSF Weill Institute for Neurosciences University of California, San Francisco San Francisco California USA; ^2^ Department of Neurology Northwestern University Chicago Illinois USA; ^3^ Department of Neurology University of Pennsylvania Philadelphia Pennsylvania USA; ^4^ Department of Neurology Ruhr University Bochum Bochum Germany; ^5^ Sutter East Bay Medical Group Lafayette California USA; ^6^ Icahn School of Medicine at Mount Sinai New York New York USA; ^7^ Texas Tech University Health Sciences Center Amarillo Texas USA; ^8^ Division of Neurology, Department of Medicine, St Michael's Hospital University of Toronto Toronto ON Canada; ^9^ Li Ka Shing Knowledge Institute University of Toronto Toronto ON Canada; ^10^ Department of Neurology Yale University New Haven Connecticut USA; ^11^ Department of Neurology Kaiser Permanente San Francisco San Francisco California USA; ^12^ Department of Neurology Swedish Medical Center Seattle Washington USA; ^13^ Department of Neurology Columbia University Irving Medical Center New York New York USA; ^14^ Department of Neurology University of Michigan Ann Arbor Michigan USA; ^15^ Rocky Mountain MS Center Salt Lake City Utah USA; ^16^ Buck Institute for Research on Aging Novato California USA

## Abstract

**Objective:**

Postpartum, patients with multiple sclerosis (MS) and neuromyelitis optica spectrum disorder (NMOSD) have increased risk for disease activity. Anti‐CD20 IgG1 monoclonal antibodies (mAb) are increasingly used as disease‐modifying therapies (DMTs). Patients may wish to both breastfeed and resume DMT postpartum. This study aimed to determine the transfer of anti‐CD20 IgG1 mAbs, ocrelizumab, and rituximab (OCR/RTX), into mature breastmilk and describe maternal and infant outcomes.

**Methods:**

Fifty‐seven *cis*‐women receiving OCR/RTX after 59 pregnancies and their infants were enrolled and followed up to 12M postpartum or 90 days post‐infusion. Breastmilk was collected pre‐infusion and serially up to 90 days and assayed for mAb concentration. Medical records and patients' questionnaire responses were obtained to assess neurologic, breastfeeding, and infant development outcomes.

**Results:**

The median average concentration of mAb in breastmilk was low (OCR: 0.08 μg/mL, range 0.05–0.4; RTX: 0.03 μg/mL, range 0.005–0.3). Concentration peaked 1–7 days post‐infusion in most (77%) and was nearly undetectable after 90 days. Median average relative infant dose was <1% (OCR: 0.1%, range 0.07–0.7; RTX: 0.04%, range 0.005–0.3). Forty‐three participants continued to breastfeed post‐infusion. At 8–12 months, the proportion of infants' growth between the 3rd and 97th World Health Organization percentiles did not differ for breastfed (36/40) and non‐breastfed (14/16, *p* > 0.05) infants; neither did the proportion with normal development (breastfed: 37/41, non‐breastfed: 11/13; *p* > 0.05). After postpartum infusion, two mothers experienced a clinical relapse.

**Interpretation:**

These confirm minimal transfer of mAb into breastmilk. Anti‐CD20 mAb therapy stabilizes MS activity before conception to the postpartum period, and postpartum treatments appears to be safe and well‐tolerated for both mother and infant.

## Introduction

The safe and effective management of neurological disease during gestation and lactation represents major unmet treatment goals across many neurological conditions. Both multiple sclerosis (MS) and neuromyelitis optica spectrum disorder (NMOSD) are more prevalent in females than in males, with onset often occurring during childbearing years.^1^ Stabilization of inflammatory activity while planning a pregnancy, during gestation, and postpartum where a heightened risk of inflammatory activity has been reported,^2^, ^3^, ^4^ requires appropriate selection and timing of disease‐modifying therapies (DMTs). Anti‐CD20 IgG1 monoclonal antibodies (mAbs), including ocrelizumab (OCR) and rituximab (RTX), are increasingly used to treat neurologic and other inflammatory conditions. The prolonged period of B‐cell depletion expected after elimination from the bloodstream, and lack of IgG1 placental transfer during the first trimester,^5^ have led to the use of these mAbs in the pregnancy planning period. Their rapid onset of effect is attractive for reducing the potential for postpartum rebound activity as well. Questions remain, however, about the safety of mAbs during lactation. This is all the more relevant in MS, where breastfeeding benefits general maternal and infant health, supports maternal–infant bonding and the maternal role, and also appears to protect against relapses.^6^ Reassuringly, the large molecular size, low lipid solubility and low oral bioavailability of IgG1 mAbs likely result in minimal breastmilk transfer and absorption by infant through breastfeeding.^7^ Two case studies,^8^, ^9^ a case series,^10^ and a small prospective cohort study^11^ have suggested minimal transfer of RTX in breastmilk with no biological impact on breastfed infants; however, larger numbers are required to confirm these findings. This prospective, multicenter cohort study aimed to evaluate the transfer of OCR and RTX into breastmilk, as well as growth, development, and immune health of infants whose mothers were treated with anti‐CD20 mAbs after delivery.

## Methods

### Study design and population

This prospective study enrolled international patients with a clinical diagnosis of MS or NMOSD receiving OCR or RTX clinically prescribed by their treating neurologist while breastfeeding or weaning and who were willing to provide breastmilk samples. All participants were enrolled in the University of California, San Francisco (UCSF) Pregnancy Registry, Infants, and Milk/Serum Analysis (PRISMA) study, which includes patients followed at the UCSF and other MS centers if they sought enrollment in the study. The samples included in this analysis were collected between November 2017 and December 2021, from participants referred from 10 MS centers in the United States and Germany. A combination of maternal records (59 pregnancies), infant records (55 total pregnancies, 56 infants), and breastmilk samples (51 pregnancies, 53 treatment cycles) was collected. A subset of the 29 women (30 pregnancies) receiving ocrelizumab were enrolled in an investigator‐initiated sub‐study sponsored by Genentech. Among the 59 pregnancies, only nine in which women received rituximab were previously reported.^11^

### Standard protocol approvals, registrations, and patient consent

The UCSF Institutional Review Board (IRB) approved this study (17‐22422). Under the same IRB, a sub‐study of ocrelizumab in breastmilk was sponsored by Genentech as an investigator‐initiated study (IIS) and listed on clinicaltrials.gov, NCT04387110. Written informed consent was obtained from all the enrolled participants in their preferred language by the UCSF study team.

### Maternal outcomes

Maternal medical records were reviewed to capture demographic data, medical history, mAb infusion date(s)/time and dose(s), and neurological data during the 12 months (M) before conception and up to 12M after delivery (or 90 days from the time of first maternal infusion, if occurring after 12M postpartum). Questionnaires were completed prospectively by participating mothers to provide pertinent pregnancy information, including medications used before and during pregnancy, pregnancy complications, MS or NMOSD relapses, infections, infusion reactions, and breastfeeding status. Telephone interviews were used to supplement missing information as needed. Clinical relapses, defined as new or worsening neurologic symptoms for ≥24 h in the absence of infection, were prospectively collected using the patient‐reported assessing relapses in MS Questionnaire at 1, 4, 8, and 12M postpartum and corroborated by the treating neurologist's note. When Expanded Disability Status Scale (EDSS) score was not explicitly included in the treating neurologist's note, this was extrapolated from the neurologic examination, documented symptoms, and reported ambulatory abilities by a neurologist (R.B.), blinded to the timing of the neurological exam relative to pregnancy.

### Infant outcomes

Infant medical records were reviewed to record delivery outcomes, immunizations, growth, laboratory results, infections, and developmental concerns. Weight, length, and head circumference recorded in medical records from delivery to 12M of age were plotted on the World Health Organization (WHO) clinical growth charts and reported as percentiles for age. When available, clinically assessed CD19 count and immunoglobulin levels from cord blood or peripheral blood were collected for infants with potential exposure to maternal anti‐CD20 therapy either in utero or during lactation. The Ages and Stages Questionnaire Version 3 (ASQ3) was completed prospectively by parents at 2, 4, 6, 8, 10, and 12M of age (when rituximab was received, ASQ3 was given at only 4, 8, and 12M). Due to the low number, survey timing was not adjusted for prematurity. The ASQ3 evaluates five domains of child development (communication, gross motor, fine motor, problem solving, and personal–social). Each domain is scored and categorized as “below cutoff”, “monitoring range”, and “above cutoff”. Both the “monitoring range” and “above cutoff” are considered on‐schedule development. “Below cutoff” suggests delayed development and indicates need for professional assessment.

### Breastmilk collection

For most participants, serial breastmilk samples were systematically collected before infusion (drug‐naïve) and at 8 and 24 h post‐infusion, in addition to 7, 18–21, 30, 60, and 90 days after one or two OCR (300 or 600 mg) or RTX (500 or 1000 mg) infusion(s) at the first and/or second postpartum treatment cycle. During an initial period of study feasibility (November 2017 to April 2019), four participants provided single‐ or two‐timepoint samples, and another five participants provided serial samples; these were previously reported^11^ and the results were included here with the current samples to provide an overview of the entire cohort and sufficient power. Between April 2019 and December 2021, 42 participants provided serial samples after 44 treatment cycles, and 2 participants who were actively weaning or enrolled post‐infusion provided single‐ or two‐timepoint samples.

### Quantification of anti‐CD20 mAb in breastmilk

To determine the breastmilk concentration of anti‐CD20 mAb therapies, fluorescent enzyme‐linked immunosorbent assay (ELISA) was used. Samples provided by participants treated with OCR were analyzed with ELISA by Syneos Health Laboratories (Richmond, VA), using affinity‐purified goat anti‐ocrelizumab polyclonal antibody as coat and a goat anti‐IgG1 antibody conjugated to horseradish peroxidase as detection. Samples provided by participants treated with RTX were analyzed by Marin Biologic Laboratories (Novato, CA) with the Eagle Biosciences rituximab ELISA Kit, using methods previously reported.^11^

Anti‐CD20 breastmilk concentrations were measured in duplicate for each sample and averaged, and when replicates of a sample were analyzed across several assays, these replicates were averaged.

### Statistical analysis

Descriptive statistics were used to characterize the cohort and infant outcomes. Then, to evaluate breastmilk parameters, for the participants who provided serial (at least 3) samples, the breastmilk concentrations of mAb were analyzed using R software (4.2.2) with pharmacokinetic methods (Ubiquity 2.0.0 non‐compartmental analysis package^12^). Two assumptions were made to conduct this analysis. First, pre‐infusion concentrations were assumed to be 0 μg/mL. Second, concentrations below the detection threshold for both OCR and RTX (0.16 and 0.01 μg/mL) were assumed to be 0.0795 and 0.005 μg/mL, respectively. Applying the trapezoidal rule, the area under the breastmilk concentration‐time curves (AUCs) was calculated for each sample set. The average concentration in breastmilk (C_AVE_) was calculated by dividing the AUC by the number of days from infusion at the time of last collection for each individual set. The maximum concentration (C_MAX_) and the time of C_MAX_ were also reported.

Using methods described by Bennett,^13^ the absolute average and maximum OCR and RTX doses to the infant in a 24‐h period and the relative infant doses (RIDs) were calculated. Calculation of the absolute average and maximum dose, as well as the RID assumes the infant will ingest an approximately 0.15 L/kg/d of breastmilk within a 24‐h period. The absolute average infant dose over 24 h is determined by multiplying the C_AVE_ by 0.15 L/kg/d, while the maximum absolute infant dose uses C_MAX_. The RID estimates an infant's potential exposure to a drug via lactation as a percentage of the maternal dose over a 24‐h period and is calculated by taking the C_AVE_ in mg/L, multiplying by 0.15 L/kg/d of breastmilk and by the maternal weight at the time of infusion, and then dividing by the maternal dose. The RID was also calculated using C_MAX_ to determine the maximum RID observed. As established by Bennett,^13^ a RID less than 10% is generally safe for an infant to breastfeed, although the drug toxicity should also be considered on a case‐by‐case basis.

Across the participants who provided serial samples, median and range were calculated for each of these measures. Participants who provided 1–2 samples were not included in these calculations; however, they were compared to those of the serially collected samples and contributed to our overall assessment of anti‐CD20 mAb transfer into breastmilk.

In relation to infant outcomes, Fisher's exact test (JMP Pro 17.0.0 statistical software package) was used to assess the difference in proportion of infants breastfed post‐maternal infusion versus not with normal growth and development (categorical) at 8–12M, reflecting methods outlined by Matro et al. 2018.^14^ Statistical significance threshold was set at p < 0.05. Maternal clinical outcomes were compared descriptively.

## Results

### Demographics and clinical characteristics

Fifty‐five *cis*‐women with relapsing–remitting MS and two *cis*‐women with NMOSD enrolled in the PRISMA study. Altogether, these 57 participants provided data corresponding to 59 pregnancies, including 33 pregnancies with postpartum treatment with OCR and 26 pregnancies with postpartum treatment with RTX. The mean age was 33.4 (SD 3.9) years, with low disability (median EDSS of 1.5) and median disease duration of 5.1 (range 0.1–14.0) years (Table 1). Among these 59 pregnancies, anti‐CD20 mAbs were also used in the year prior to conception for 33 (*n* = 27 ≤ 6 M prior to conception). The median time between delivery and postpartum infusion in the samples provided was 2.6 M (range 0.1–36.0). Among the 59 total pregnancies (*n* = 58 singleton, *n* = 1 twin), 51 were term pregnancies and 8 pregnancies were preterm (<37 weeks gestation).

**Table 1:** Demographic and clinical characteristics of the cohort (*n* = 59 pregnancies in 57 women).

Characteristic	Pregnancies (*n* = 59)		
	Ocrelizumab (*n* = 33)	Rituximab (*n* = 26)	Entire cohort (*n* = 59)
Maternal age at conception, mean years (SD)	33.7 (3.6)	33.8 (4.3)	33.4 (3.9)
Race, *n* (%)			
White	32 (97.0)	18 (69.2)	50 (84.7)
Asian	1 (3.0)	5 (19.2)	6 (10.2)
African American	0	2 (7.7)	2 (3.4)
Other	0	1 (3.8)	1 (1.7)
Education, median years (min–max)	16 (12–22)	17 (12–23)	
Diagnosis at enrollment, *n*			
RRMS	33^a^	24^b^	57^a^ ^,^ ^b^
NMOSD	0	2	2
Disease duration, median years (min–max)	5.1 (0.4–14.0)^a^	4.9 (0.1–13.0)	5.1 (0.1–14.0)^a^
Preconception EDSS, median (min–max)	1.5 (0–2.5)^c^	1.5 (0–4.0)^d^	1.5 (0–4.0)^c^ ^,^ ^d^
DMT prior to conception, *n* (%)			
Anti‐CD20 mAb	18 (54.5)	15 (57.7)	33 (55.9)
Other DMT	9 (27.3)	7 (26.9)	16 (27.1)
DMT naive	6 (18.2)	4 (15.4)	10 (16.9)
If treated with anti‐CD20 mAb <12 M of conception			
Duration of treatment prior to conception, median M (min–max)	8.9 (1.6–31.6)	16.6 (2.2–42.3)	14.9 (0.5–42.3)
Infusion to conception, median M (min–max)	4.1 (0.7–10.2)	2.3 (−0.3–7.3)^e^	3.5 (−0.3–10.2)^e^
Delivery to first postpartum infusion, median M (min–max)	4.3 (0.1–36.0)	1.9 (0.2–12.1)	2.6 (0.1–36.0)
Postpartum anti‐CD20 mAb dose, *n*			
1 × half dose^f^	2	2	4
2 × half dose^f^	17	9	26
1 × full dose^g^	14	11	25
2 × full dose^g^	0	4	4
No. of infants breastfed (≥2 weeks) post‐infusion, *n* (%)	24 (72.7)	19 (70.4)^h^	43 (71.7)^h^
Exclusively, *n*	17	10	27
Partially, *n*	7	9	16

DMT, Disease‐modifying therapy, EDSS, Expanded Disability Status Scale; M, month(s); mAb, monoclonal antibody; NMOSD=neuromyelitis optica spectrum disorders; RRMS, relapsing–remitting multiple sclerosis; SD, standard deviation.

^a^ *n* = 32 with diagnosis before pregnancy and *n* = 1 with postpartum onset in the ocrelizumab treatment group.

^b^ *n* = 1 diagnosis was amended to myelin oligodendrocyte glycoprotein antibody disorder 3 years after completing participation in the rituximab treatment group.

^c^ *n* = 31 (*n* = 1 with postpartum onset, *n* = 1 without exam) in the ocrelizumab treatment group.

^d^ *n* = 22 (*n* = 1 with onset in pregnancy, *n* = 2 with NMOSD, *n* = 1 without exam) in the rituximab treatment group.

^e^ *n* = 1 with infusion at 7 weeks and 1 day in the rituximab treatment group.

^f^ “Half” dose is 300 mg (ocrelizumab) or 500 mg (rituximab).

^g^ “Full” dose is 600 mg (ocrelizumab) or 1000 mg (rituximab).

^h^ Denominator of *n* = 27 infants (one twin pregnancy) in the rituximab treatment group.

### Maternal clinical outcomes

#### Participants with MS

**Figure 1:**
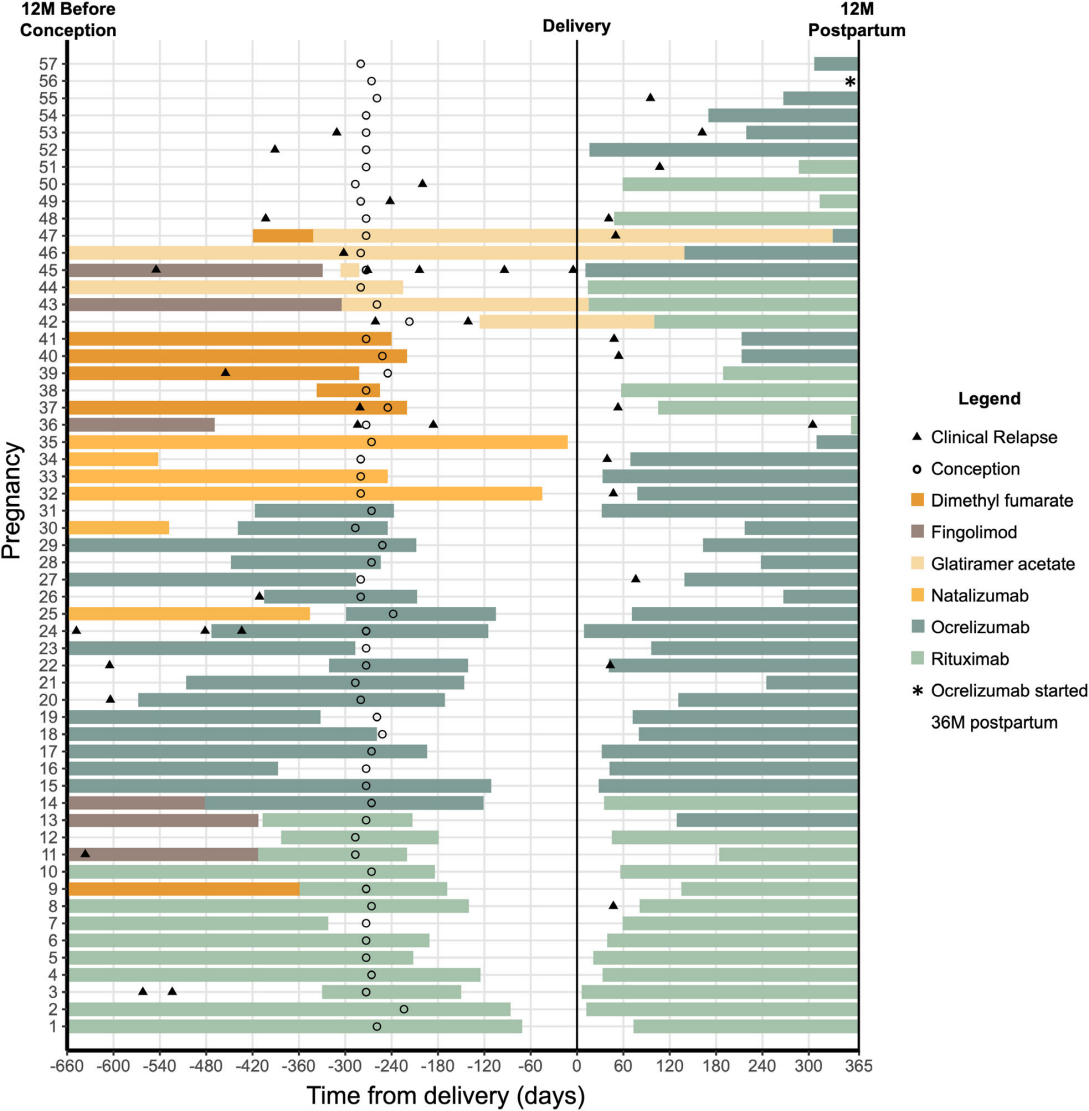
Timing of maternal relapse and DMT use relative to pregnancy in women with MS (*n* = 57 pregnancies). Each line on the *y*‐axis indicates a single pregnancy. The *x*‐axis indicates observation period from 12 months before conception, through pregnancy, and up to 12 months postpartum. The color of each line indicates maternal DMT at a given timepoint; anti‐CD20 and natalizumab treatment were graphed with extension to 6M and 1M past the last infusion, respectively. Conception is indicated by a circle. Relapse is indicated by a triangle and was assessed from 12 months before conception to 12 months after delivery.

Clinical relapses and their association with DMT use from 12M before conception through 12M postpartum are visualized in Figure 1. Altogether, in the 12M before conception, a relapse occurred in 15/57 pregnancies; in five of these, the patient was untreated at the time of the relapse and subsequently started anti‐CD20 DMT (one of whom relapsed 1M later after receiving only one half of the ocrelizumab start dose, 300 mg). During pregnancy, a relapse occurred in 5/57 pregnancies (*n* = 3: no DMT, one of whom subsequently started glatiramer acetate during pregnancy, *n* = 1: fingolimod discontinued before conception, *n* = 1: fingolimod discontinued before conception and switched to glatiramer acetate during pregnancy, with four relapses). Postpartum, a relapse occurred in 13/57 pregnancies between delivery and first anti‐CD20 infusion. Between anti‐CD20 infusion and the 12M postpartum, one relapse occurred among 57 pregnancies, 2 days after infusion.

Focusing on patients treated using optimized windows for anti‐CD20, with treatment within 6M of conception, 1/25 experienced a relapse between infusion and delivery (1M after receiving only one half of the ocrelizumab start dose); 2/25 experienced a relapse between delivery and infusion (infusion was >5 weeks postpartum in both cases); and 1/25 experienced a relapse 2 days after infusion (i.e., before treatment was therapeutic). Of all 14 participants who were treated with anti‐CD20 within 5 weeks of delivery, 0/14 experienced a clinical relapse between delivery and 12M postpartum.

#### Participants with NMOSD

Two participants with NMOSD received anti‐CD20 therapy within 6M prior to conception. Neither experienced a relapse in the year before conception or during pregnancy. Postpartum, one relapse occurred in one pregnancy 1.5M after delivery, 14 days after anti‐CD20 therapy had been resumed.

#### Immunological parameters

Maternal infections were also prospectively collected (COVID‐19, Flu, Cold, etc.). No maternal infections requiring hospitalization were reported. Four women reported mastitis during their participation in the study; however, none of these infections coincided with breastmilk collection.

In 17 pregnancies, serum IgG, IgM, and/or IgA levels were assessed clinically postpartum and prior to maternal infusion (median 50 days after delivery, range 0–850). Among women with anti‐CD20 within 6M of conception, IgG (13/16 pregnancies), IgM (7/8 pregnancies), and IgA (14/15 pregnancies) were within normal range. The three abnormal postpartum IgG levels were: 275, 589, and 652 mg/dL (normal range: 672–1,760 mg/dL). Among the women with low postpartum IgG postpartum, two women had been treated with OCR for ≥12M prior to conception and one participant was DMT naïve.

### Anti‐CD20 mAb concentration in breastmilk

**Figure 2:**
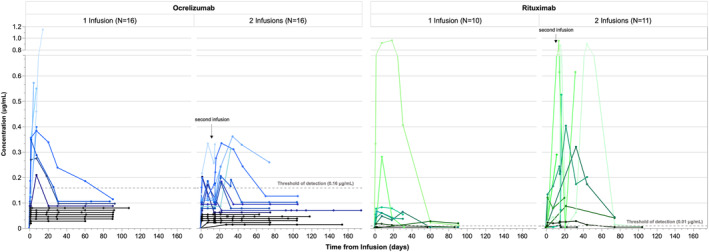
Anti‐CD20 mAb in breastmilk (*n* = 32 Ocrelizumab and *n* = 21 Rituximab treatment cycles). Breastmilk concentration (μg/mL) in 51 pregnancies up to 174 days post‐infusion. Lines connect samples from a given individual during a single collection period. Pre‐infusion concentration was assumed to be 0 μg/mL. Samples below the detection threshold (ocrelizumab: 0.16 μg/mL, rituximab: 0.01 μg/mL) were plotted arbitrarily at concentrations under the threshold for visual clarity. Pregnancies with no detectable concentration of anti‐CD20 mAb in any sample are plotted in black.

A total of 393 breastmilk samples were collected after 53 treatment cycles in 51 pregnancies. The concentration of OCR and RTX in breastmilk measured in all samples is displayed in Figure 2. For the 49 treatment cycles (47 pregnancies) providing three or more serial milk samples, median average concentration of mAb was low at 0.08 μg/mL for OCR (range 0.05–0.4) and 0.03 μg/mL for RTX (range 0.005–0.3). In sample sets with detectable concentrations, peak concentration occurred in most (77%) 1–7 days post‐infusion and was nearly undetectable by 90 days post‐infusion, with a median maximum concentration 0.3 μg/mL for OCR (range 0.2–0.5 [*n* = 16 with detectable concentrations]) and 0.1 μg/mL for RTX (range 0.01–0.9 [*n* = 15 with detectable concentrations]). Based on C_AVE_, RID was 0.1% for OCR (range 0.07–0.7) and 0.04% for RTX (range 0.005–0.3) (Table 2).

**Table 2:** Minimal transfer of anti‐CD20 monoclonal antibodies into breastmilk serially collected across 43 pregnancies (*n* = 45 mAb treatment cycles).

Parameters	Ocrelizumab (*n* = 30 treatments)*			Rituximab (*n* = 15 treatments)**			
	1 × 300 mg (*n* = 4)	1 × 600 mg (*n* = 10)	2 × 300 mg (*n* = 16)	1 × 500 mg (*n* = 2)	1 × 1000 mg (*n* = 6)	2 × 500 mg (*n* = 5)	2 × 1000 mg (*n* = 2)
	Median (min–max)						
AUC_0‐Tmax_, μg·d/mL	4.3 (0.07–7.2)	7.7 (2.9–20.1)	8.2 (2.1–18.6)	1.1 (0.8–1.3)	2.1 (0.3–28.1)	0.7 (0.2–9.7)	12.3 (9.7–14.8)
T_MAX_, d	49 (1–91)	90 (7–107)	90 (14–174)	58 (30–86)	73 (21–90)	31 (16–104)	74 (44–104)
C_AVE_, μg/mL	0.08 (0.3–7.2)	0.1 (0.1–0.4)	0.08 (0.05–0.3)	0.03 (0.01–0.04)	0.04 (0.005–0.3)	0.02 (0.007–0.3)	0.2 (0.1–0.2)
C_MAX_, μg/mL^a^	0.4	0.3 (0.2–0.5)	0.2 (0.2–0.4)	0.04 (0.02–0.06)	0.07 (0.005–1.0)	0.1 (0.01–0.9)	0.6 (0.4–0.9)
Time of C_MAX_, d^a^ ^,^ ^b^	7	7 (1–7)	7 (1–19)	4 (0–7)	7 (0.3–30)	2 (0.3–18)	11 (7–14)
RID from C_AVE_, %	0.2 (0.2–0.7)	0.1 (0.1–0.6)	0.1 (0.07–0.3)	0.06 (0.02–0.1)	0.05 (0.005–0.3)	0.01 (0.007–0.3)	0.09 (0.06–0.1)
RID from C_MAX_, %^a^	1.4	0.6 (0.3–0.8)	0.5 (0.3–0.8)	0.08 (0.03–0.1)	0.07 (0.005–0.8)	0.1 (0.03–0.9)	0.3 (0.3–0.4)
Absolute infant dose by C_AVE_, mg/kg/d	0.01 (0.01–0.03)	0.01 (0.01–0.06)	0.01 (0.008–0.04)	0.004 (0.001–0.007)	0.006 (0.0007–0.05)	0.003 (0.001–0.05)	0.03 (0.02–0.03)
Absolute infant dose by C_MAX_, mg/kg/d^a^	0.06	0.05 (0.03–0.08)	0.03 (0.03–0.05)	0.006 (0.003–0.009)	0.01 (0.0007–0.01)	0.02 (0.002–0.1)	0.1 (0.06–0.1)

The AUC, average concentration, maximum concentration, and RID of both ocrelizumab and rituximab is presented.

AUC_0‐Tmax_, area under the drug–concentration–time curve in milk; C_AVE_, average drug concentration over the interval; C_MAX,_ maximum drug concentration measured; RID, relative infant dose, T_MAX_, maximum time of milk collection.

^a^ Only calculated for patients with a detectable concentration (above the assay threshold).

^b^ Calculated relative to most recent infusion (first or second).

* ELISA conducted by Syneos Health Laboratories.

** ELISA conducted by Marin Biologic Laboratories.

Among the participants who provided a single or two samples, the observed concentrations were similar to those of patients with serial samples sets apart from one outlier with a concentration of 1.15 μg/mL at 14 days post‐infusion (RID 3.4%). This outlying concentration and similar sample sets ending on a peak in concentration were collected at the time of weaning and subsequent samples were not available; thus the rate of decline from peak concentration could not be calculated.

### Infant outcomes

#### Neonatal outcomes relating to preconception maternal treatment

**Figure 3:**
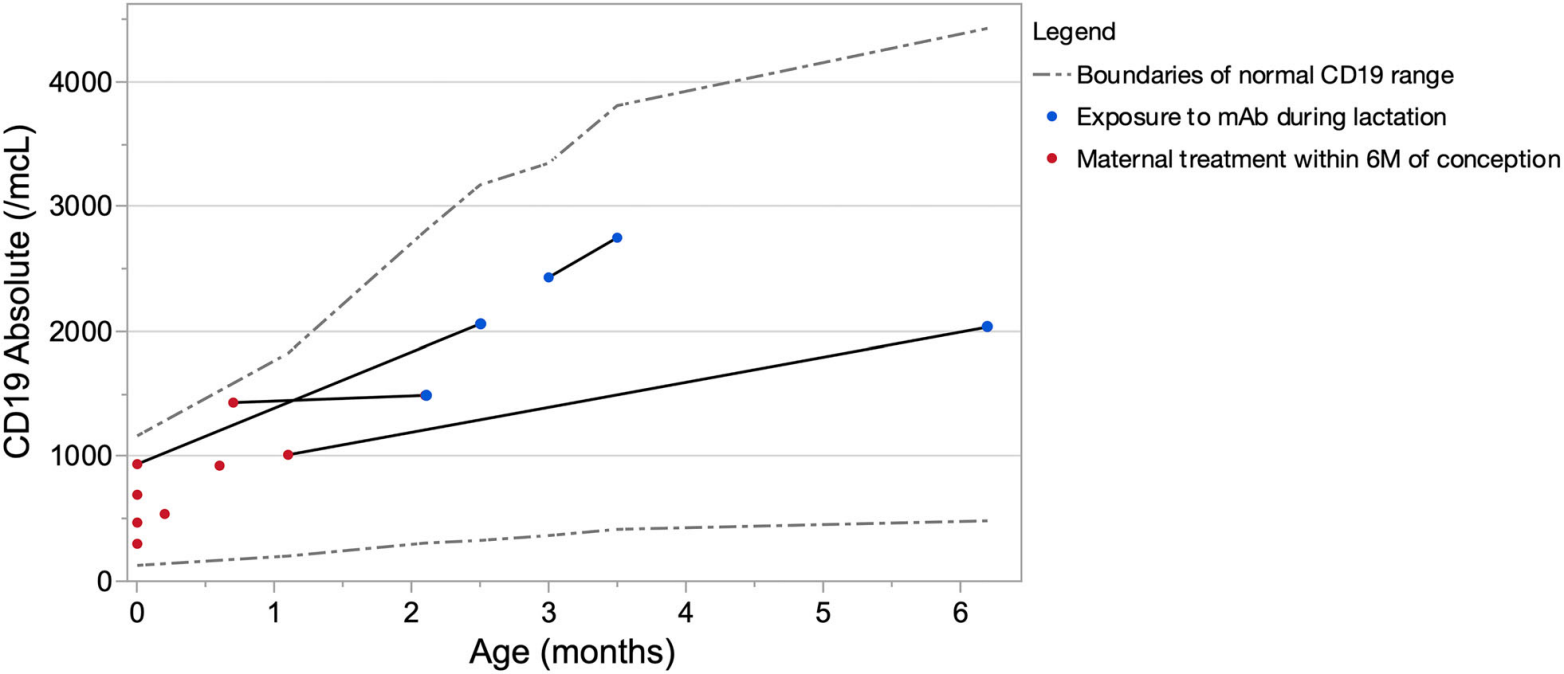
Infant CD19 levels (*n* = 9 infants). Individual lines connect collection timepoints provided by a given infant. Red timepoints indicate sample collection in infant wih maternal treatment with mAb <6 months before conception (*n* = 8). Blue timepoints indicate sample collection after infant exposure to mAb via lactation (*n* = 4, breastfeeding after maternal postpartum infusion). Boundaries indicating the 10th–90th percentiles for absolute CD19+ cells among healthy infants (300–2000 CD19+ cells/μL aged 0–3 months, 430–3000 CD19+ cells/μL 3–6 months) are plotted as a gray dashed line.

Eight infants with maternal anti‐CD20 treatment within 6M of conception and one infant with maternal infusion at 7 weeks and 1 day underwent clinical evaluation of cord and/or peripheral blood for IgG and CD19 levels; all were within normal range (Fig. 3).^15^, ^16^ All infants were exclusively breastfeeding at the time of peripheral blood collection.

#### Postnatal outcomes relating to postpartum maternal infusion

**Figure 4:**
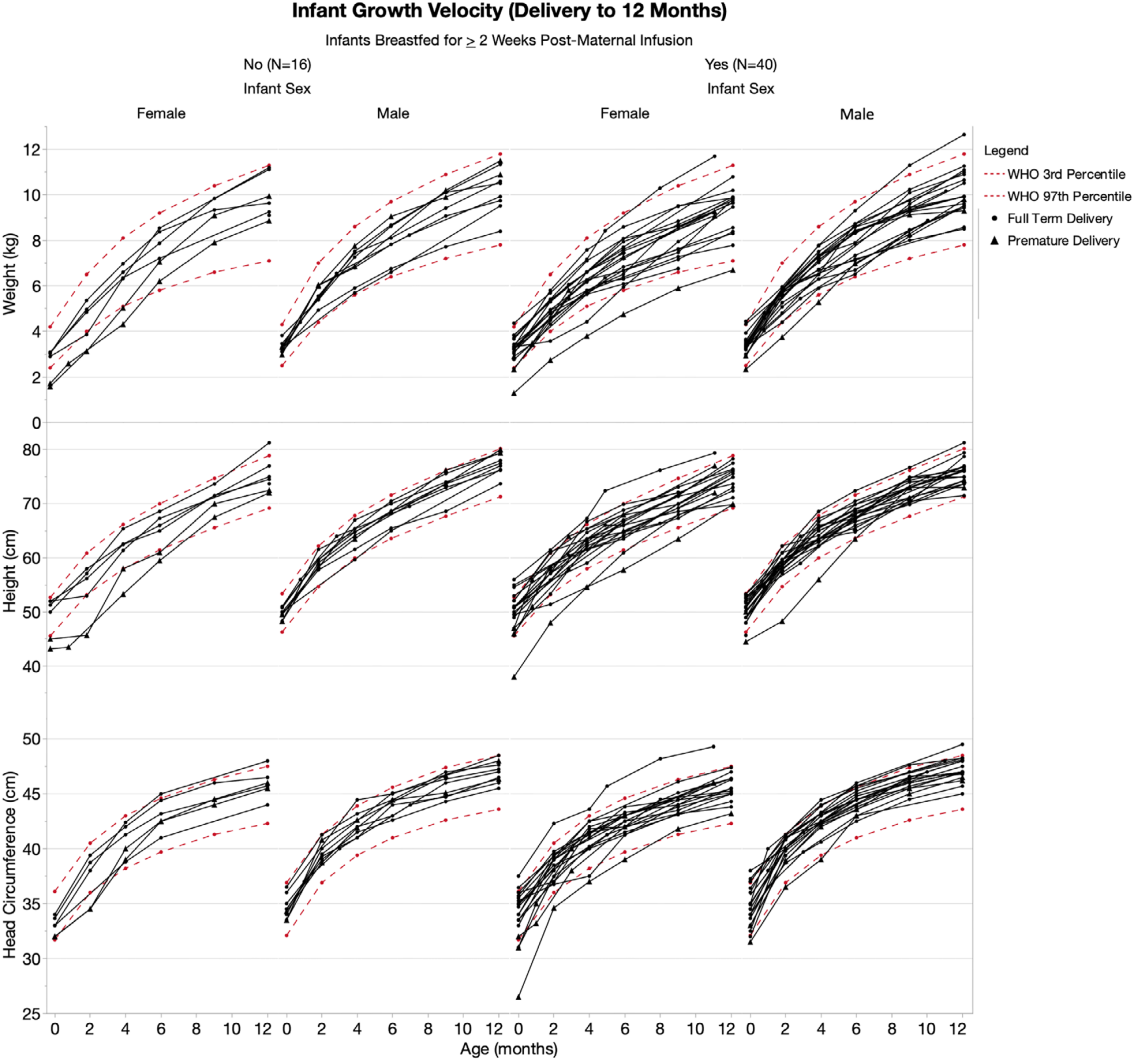
Infant growth velocity. Weight, length, and head circumference from birth to 12 months (*n* = 56). Normal percentiles, defined by the WHO as 3rd‐97th percentiles, are graphed in red. Each black line corresponds to a given infant relative to age indicated on the *x*‐axis. There were no statistical differences in growth between breastfed and non‐breastfed infants (weight, height, and head circumference: *p* > 0.05 on Fisher's exact test).

**Figure 5:**
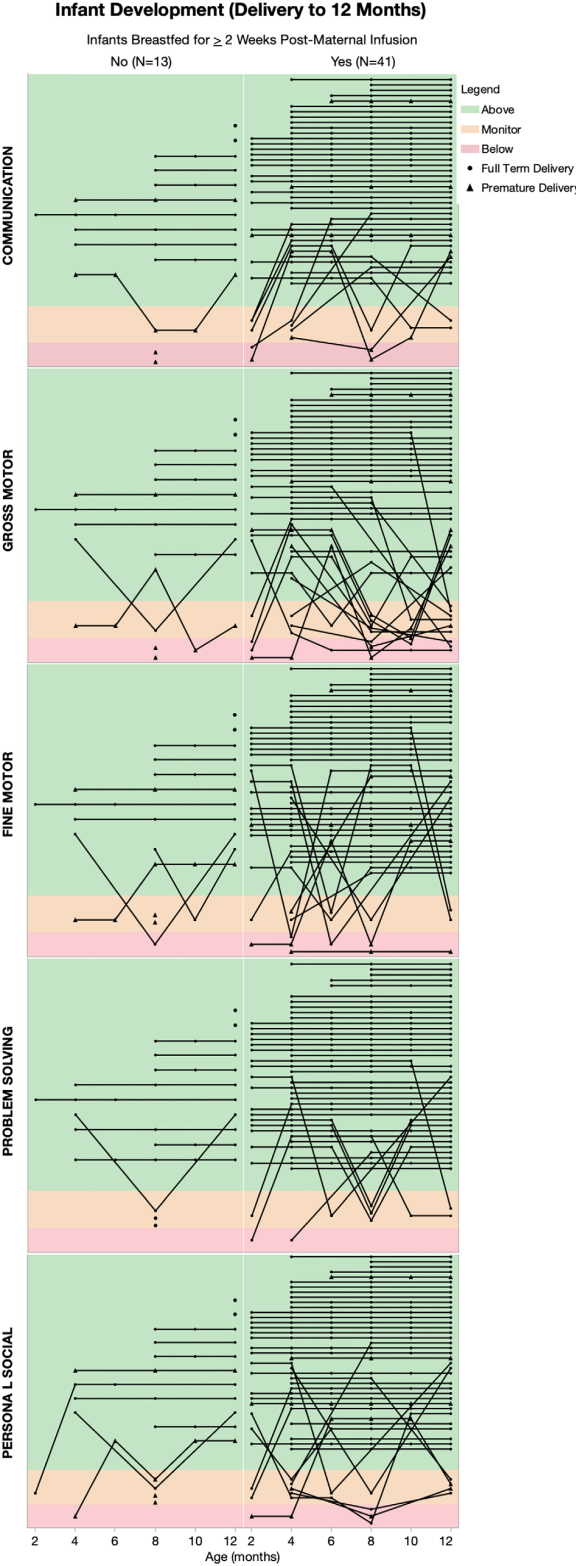
Infant development ASQ scores in five domains of development (*n* = 54 infants). “Above cutoff” and “monitoring range” indicated on schedule development. Lines connect timepoints for a given infant. Surveys were not adjusted for gestational age at delivery. There were no statistical differences in growth between breastfed and non‐breastfed infants (*p* > 0.05 for each domain on Fisher's exact test).

After maternal postpartum anti‐CD20 infusion, participants continued to breastfeed for at least 14 days in 43 pregnancies (*n* = 27 exclusively, *n* = 16 partially); median duration of breastfeeding post‐infusion and up to 12M postpartum was 6.4 M (range 0.3–11.7). When comparing the breastfed versus non‐breastfed infants, there were no differences in the proportion of infants whose 8‐12 M measurements fell within the WHO standards for normal growth (i.e., 3rd and 97th percentiles, *p* > 0.05 for weight, height, and head circumference; Figure 4). In total, 36/40 breastfed infants had normal measurements across all three parameters, as did 14/16 non‐breastfed infants (*p* > 0.05). Additionally, there were no differences in the proportion of breastfed (37/41 with normal development) versus non‐breastfed infants (11/13 with normal development) in ASQ3 development scores up to 8–12M (Fig. 5 **),** *p* > 0.05 in all domains (communication, gross motor, fine motor, problem solving, and personal–social). From the clinical records available for 56 infants (including both breastfed and non‐breastfed), there were minor but nonserious infections which are common in infancy (e.g., upper respiratory infection, otitis media, COVID‐19 infection, common cold; Supplementary Table S1); there were no infant hospitalizations due to infection. Routine vaccinations were given in 53/56 per CDC guidelines^17^; three parents deferred the live rotavirus vaccination for their infants.

Four infants between the ages of 2.1 and 6.2M who were breastfed after maternal infusion underwent clinical evaluation of peripheral blood for IgG and CD19 levels, all were within the normal range (Fig. 3).

## Discussion

In this large prospective multicenter international study of women with MS and NMOSD treated clinically with anti‐CD20 mAbs (OCR and RTX), minimal concentration of mAb was found in mature breastmilk, corresponding to a very low RID, well below the theoretically acceptable threshold. Infants who were subsequently breastfed did not differ in growth or development from infants who were weaned, and immunological parameters were normal when measured, suggesting negligible uptake of any residual undigested anti‐CD20 IgG drug molecules into the infant circulation.^18^ Additionally, there were almost no maternal relapses postpartum after treatment, and no relapses during the entire preconception to postpartum time period in women completing treatment within 6M of conception and 1M postpartum.

Several aspects of the findings, reassuringly, support the safety of breastfeeding during anti‐CD20 treatment. First, similar trends were observed for both OCR and RTX, including RID (<1%) and time to peak (1–10 days). Second, the findings from the preliminary cohort of RTX patients were reaffirmed in the current larger dataset. Third, the highest concentrations were noted in the mothers who were actively weaning, which is consistent with the physiology of the mammary gland when discontinuing breastfeeding.^19^ Finally, these findings are in alignment with other reports of anti‐CD20 transfer in breastmilk.^8^, ^9^, ^10^ While this dataset was comprehensive, large, and multicentric, limitations included the low number of infants with blood drawn clinically for CD19 count and immunoglobulins; a consequence perhaps of the COVID‐19 pandemic as well as general reassurance by treating pediatricians about the low likelihood of breastmilk transfer and low risk to the infant. In fact, both the American College of Rheumatology and the American Gastroenterological Association already support breastfeeding with mAb treatment.^20^, ^21^

Historically, about one third of all women with MS relapse postpartum,^4^, ^22^ and over 50% develop new lesions on brain MRI.^23^, ^24^ Similarly, women with NMOSD also face heightened risk for postpartum relapse.^25^ This risk depends on baseline disease activity, age, and treatment parameters of the cohort. In the current study of women treated with anti‐CD20 therapy, there were very few relapses; none relapsed before or during pregnancy when treated within 6M of conception. Further, none relapsed postpartum when infused within 5 weeks of delivery. Historically, when interpreting effects of breastfeeding on postpartum relapse risk, there was concern that breastfeeding reflected a more benign disease course since women with higher relapse risk may be advised to forego breastfeeding and resume treatment early.^26^ More recently, however, there has been a trend toward more actively treating women before and after pregnancy, even if breastfeeding. In fact, use of anti‐CD20 mAbs in this period could reflect concern about a patient's elevated relapse risk. Altogether, the low risk of relapses observed likely reflects the true effect of these high efficacy mAbs, which have little therapeutic lag in the postpartum period.

Clinically, these results confirm that anti‐CD20 therapy effectively reduces MS disease activity before conception through to the postpartum period, minimal transfer into breastmilk, and reassuring infant development, growth, and CD19 levels when exposed to anti‐CD20 therapies via breastfeeding. The findings may have relevance to the use of several other IgG1 mAbs in the management of pregnant and lactating women with neurological diseases, including MS, NMOSD, myasthenia gravis, and other neuroimmune conditions, as well as more common conditions such as migraines.

## Author Contributions

Annika Anderson was responsible for the acquisition and analysis of data and drafted and revised the manuscript. William Rowles, Shane Poole, Ayushi Balan, Dr. Carolyn Bevan, Dr. Rachel Brandstadter, Dr. Andrea I. Ciplea, Dr. Joanna Cooper, Dr. Michelle Fabian, Dr. Thomas W. Hale, Dr. Dina Jacobs, Dr. Mihir Kakara, Dr. Kristen M. Krysko, Dr. Erin E. Longbrake, Dr. Jacqueline Marcus, Dr. Pavle Repovic, Dr. Claire S. Riley, Dr. Andrew R. Romeo, Dr. Alice Rutatangwa, Dr. Timothy West, Dr. Kristen Hellwig, and Dr. Sara C. LaHue were responsible for the aquisition and analysis of data and revised the manuscript. Dr. Riley Bove conceptualized and supervised the study and drafted and revised the manuscript.

## Funding Information

The PI (RB) is funded through a Harry Weaver Award from the National Multiple Sclerosis Society (NMSS). Support for enrollment and analysis of patients infused with ocrelizumab (only) was provided through an investigator‐initiated study sponsored by Genentech, Inc, the manufacturer of ocrelizumab.

## Conflict of Interest

All authors’ financial disclosures are included in the attached ICMJE forms. The drugs, ocrelizumab and rituximab, are tested in this study. Genentech (a subsidiary of the Roche Group) is the manufacturer of ocrelizumab and co‐markets rituximab, a drug developed and co‐marketed by Biogen. Potential conflict of interest disclosures related to the study are outlined below: CB has received scientific advisory board fees and travel support from Genentech which manufactures and co‐markets the AIC reports speaker honoraria from Biogen which co‐markets a drug tested in this study. DJ has received research support and consulting/scientific advisory board fees from Biogen and Genentech which manufacture and co‐market drugs tested in this study. KMK is an advisory board member for Roche and has received grants from Roche which manufactures and co‐markets a drug tested in this study. KMK also reports support from an MS fellowship grant during her fellowship from Biogen, which co‐markets a drug tested in this study. EEL reports research support and consulting/advisory board fees from Genentech which manufactures and co‐markets the drugs tested in this study. PR reports consulting and/or speaking honoraria from Genentech which manufactures and co‐markets the drugs tested in this study. CSR reports consulting fees or honoraria from Genentech which manufactures and co‐markets the drugs tested in this study. TW has received honoraria for speaking and education activities through Biogen Idec which co‐markets a drug tested in this study. KH has received research support, consulting, speaking, and/or advisory board fees from Roche and Biogen which manufacture and co‐market the drugs tested in this study. RB reports research support from Biogen and Roche‐Genentech which manufactures and co‐markets the drugs tested in this study.

## Supporting information


**Table S1**.

## Data Availability

De‐identified data will be shared by the corresponding author with any qualified investigator by request.
