# Supplementary material for: Anti‐CD20 monoclonal antibody therapy in postpartum women with neurological conditions
Source: Ann Clin Transl Neurol. 2023 Sep 7;10(11):2053–64. doi: 10.1002/acn3.51893 (PMC10647007; doi:10.1002/acn3.51893)
Supplement: Supplementary file 1 — Table S1. [file ACN3-10-2053-s001.docx]

**Supplementary Table 1.** Summary of infant infections in the first 12 months of life (n=56 infants, n=55 pregnancies).

| Breastfeeding Group | Infant ID | Infant age at the time of maternal postpartum infusion (months) | No Infections Reported | Common Infections in Infancy | | | | | | | | | | | Uncommon or Severe Infections (e.g. pneumonia, fungal infection, meningitis, sepsis) |
| --- | --- | --- | --- | --- | --- | --- | --- | --- | --- | --- | --- | --- | --- | --- | --- |
|  |  |  |  | Nasopharyngitis | Upper Respiratory Tract Infection | Otitis Media | Viral Illness/Fever | Influenza | COVID-19 | Decryostenosis and/or Conjunctivitis | Urinary Tract Infection | Diaper Rash (Candida) | Oral Thrush (Candida) | GERD/esophagitis |  |
| Breastfed >2 Weeks Post-Maternal Ant-CD20 Treatment | 1 | 0.5 |  |  |  |  | 6M, 8M |  |  |  |  |  |  |  |  |
|  | 2 | 1.1 |  |  |  |  |  |  |  |  |  | 2w | 1.5M |  |  |
|  | 3 | 12.0 | X |  |  |  |  |  |  |  |  |  |  |  |  |
|  | 4 | 1.8 | X |  |  |  |  |  |  |  |  |  |  |  |  |
|  | 5 | 4.4 |  |  |  |  | 7M |  |  |  |  |  |  |  |  |
|  | 6 | 2.7 |  |  |  | 10M |  |  |  | 2w |  | 5d |  |  |  |
|  | 7 | 0.5 |  | 2M | 3M,12M |  | 11M |  |  |  |  |  |  | 3w |  |
|  | 8 | 1.6 | X |  |  |  |  |  |  |  |  |  |  |  |  |
|  | 9 | 1.8 | X |  |  |  |  |  |  |  |  |  |  |  |  |
|  | 10 | 3.4 | X |  |  |  |  |  |  |  |  |  |  |  |  |
|  | 11 | 1.9 |  | 1M |  |  | 8M |  |  |  |  |  |  |  |  |
|  | 12 | 3.3 | X |  |  |  |  |  |  |  |  |  |  |  |  |
|  | 13 | 1.3 |  |  | 8M |  | 12M |  |  |  |  |  |  |  |  |
|  | 14 | 0.7 |  |  |  |  | 10M |  | 12M |  |  |  |  |  |  |
|  | 15 | 7.8 |  |  |  |  | 4M,6M, 9M |  |  |  |  |  |  |  |  |
|  | 16 | 1.1 |  |  |  |  |  |  |  |  |  | 2M |  |  |  |
|  | 17 | 6.0 |  |  | 5M, 7M, 8M | 5M, 6M, 7M, 8M, 9M |  |  |  |  |  |  |  | 2M |  |
|  | 18 | 7.8 |  |  | 12M |  |  |  |  | 1M |  |  |  |  |  |
|  | 19 | 4.6 | X |  |  |  |  |  |  |  |  |  |  |  |  |
|  | 20 | 2.3 | X |  |  |  |  |  |  |  |  |  |  |  |  |
|  | 21 | 8.8 |  |  |  |  |  |  |  | 4M |  |  |  |  |  |
|  | 22 | 5.6 |  |  |  |  |  |  |  |  |  | 10M |  |  |  |
|  | 23 | 1.1 | X |  |  |  |  |  |  |  |  |  |  |  |  |
|  | 24 | 4.6 |  |  | 1M |  |  |  |  |  |  |  |  |  |  |
|  | 25 | 2.3 | X |  |  |  |  |  |  |  |  |  |  |  |  |
|  | 26 | 0.4 |  | 4M |  |  |  |  |  |  |  |  |  |  |  |
|  | 27 | 7.0 | X |  |  |  |  |  |  |  |  |  |  |  |  |
|  | 28 | 0.3 | X |  |  |  |  |  |  |  |  |  |  |  |  |
|  | 29 | 3.1 | X |  |  |  |  |  |  |  |  |  |  |  |  |
|  | 30 | 8.0 |  | 3M, 4M |  | 8M | 2M |  |  |  |  |  |  |  |  |
|  | 31 | 4.3 | X |  |  |  |  |  |  |  |  |  |  |  |  |
|  | 32 | 7.2 | X |  |  |  |  |  |  |  |  |  |  |  |  |
|  | 33 | 2.4 |  |  |  | 11M |  |  |  |  |  |  |  |  |  |
|  | 34 | 2.6 |  | 10M |  |  | 9M |  |  |  |  |  |  |  |  |
|  | 35 | 2.6 | X |  |  |  |  |  |  |  |  |  |  |  |  |
|  | 36 | 4.2 | X |  |  |  |  |  |  |  |  |  |  |  |  |
|  | 37 | 1.0 |  |  |  |  |  |  | 12M |  |  |  |  |  |  |
|  | 38 | 0.5 |  |  |  |  | 12M |  |  |  |  |  |  |  |  |
|  | 39 | 1.4 |  |  |  |  |  |  | 8M |  |  |  |  |  |  |
|  | 40 | 0.9 | X |  |  |  |  |  |  |  |  |  |  |  |  |
| Non-Breastfed Post-Maternal Anti-CD20 Treatment | 41 | 1.9 |  |  | 12M |  |  |  |  |  |  |  |  |  |  |
|  | 42 | 1.5 |  |  |  |  | 10M |  |  |  |  |  |  |  |  |
|  | 43 | 1.1 |  |  |  |  |  |  |  |  |  | 12M |  |  |  |
|  | 44 | 6.2 |  | 3M | 4M |  | 4.5M, 6M |  |  |  |  |  |  |  |  |
|  | 45 | 0.2 |  |  | 9M |  | 12M |  |  |  | 5M |  |  |  |  |
|  | 46 | 0.4 |  |  | 5M |  |  |  |  |  |  | 3M |  | 1M |  |
|  | 47 | 0.4 |  |  | 5M |  |  |  |  |  |  |  |  |  |  |
|  | 48 | 10.1 |  | 4M |  |  |  |  |  |  |  |  |  |  |  |
|  | 49 | 10.9 |  | 12M |  |  |  |  |  |  |  |  |  |  |  |
|  | 50 | 7.0 | X |  |  |  |  |  |  |  |  |  |  |  |  |
|  | 51 | 36 | X |  |  |  |  |  |  |  |  |  |  |  |  |
|  | 52 | 1.0 |  | 7M |  | 9M |  |  |  |  |  |  |  |  |  |
|  | 53 | 10.2 | X |  |  |  |  |  |  |  |  |  |  |  |  |
|  | 54 | 7.1 | X |  |  |  |  |  |  |  |  |  |  |  |  |
|  | 55 | 5.3 |  |  | 1M |  | 3M, 7M |  |  |  |  |  |  |  |  |
|  | 56 | 8.8 | X |  |  |  |  |  |  |  |  |  |  |  |  |
